# Towards Objective Quantification of Hand Tremors and Bradykinesia Using Contactless Sensors: A Systematic Review

**DOI:** 10.3389/fnagi.2021.716102

**Published:** 2021-10-25

**Authors:** Augusto Garcia-Agundez, Carsten Eickhoff

**Affiliations:** AI Lab, Brown Center for Biomedical Informatics, Brown University, Providence, RI, United States

**Keywords:** bradykinesia, Parkinson's disease, UPDRS, leap motion, contactless

## Abstract

Assessing the progression of movement disorders such as Parkinson's Disease (PD) is key in adjusting therapeutic interventions. However, current methods are still based on subjective factors such as visual observation, resulting in significant inter-rater variability on clinical scales such as UPDRS. Recent studies show the potential of sensor-based methods to address this limitation. The goal of this systematic review is to provide an up-to-date analysis of contactless sensor-based methods to estimate hand dexterity UPDRS scores in PD patients. Two hundred and twenty-four abstracts were screened and nine articles selected for analysis. Evidence obtained in a cumulative cohort of *n* = 187 patients and 1, 385 samples indicates that contactless sensors, particularly the Leap Motion Controller (LMC), can be used to assess UPDRS hand motor tasks 3.4, 3.5, 3.6, 3.15, and 3.17, although accuracy varies. Early evidence shows that sensor-based methods have clinical potential and might, after refinement, complement, or serve as a support to subjective assessment procedures. Given the nature of UPDRS assessment, future studies should observe whether LMC classification error falls within inter-rater variability for clinician-measured UPDRS scores to validate its clinical utility. Conversely, variables relevant to LMC classification such as power spectral densities or movement opening and closing speeds could set the basis for the design of more objective expert systems to assess hand dexterity in PD.

## 1. Introduction

Parkinson's Disease (PD) is a movement disorder caused by the degeneration of the dopaminergic neurons of the *substantia nigra pars compacta*, a reduction of striatal dopamine, and is characterized by the potential presence of Lewy bodies (Jameson, 2018). PD requires constant monitoring to track progression and perform therapeutic adjustments. Monitoring is currently performed with questionnaires such as the Unified PD Rating Scale (UPDRS) (Goetz et al., 2008).

UPDRS rates different aspects of PD through visual observation of a series of tasks. These tasks are designed to monitor, among others, the most important symptoms of PD, also known as cardinal signs: resting tremors, asymmetry, bradykinesia, and a positive response to dopaminergic replacement therapy. In the case of hand dexterity, these tasks are related to bradykinesia and hand tremors, performing tasks such as finger tapping. UPDRS then rates these tasks on scales from zero (no symptoms) to four (patient is unable to perform the task), through visual observation. While the criteria to identify zeroes and fours are mostly clear, intermediate scores are considerably more ambiguous, which irrevocably leads to sensibility and reliability problems (Patrick et al., 2001). UPDRS is commonly complemented with patient diaries, which, although helpful, can also be biased by the subjective view of the patient (Hauser et al., 2004). The fact that this ambiguity introduces variability in assessments is well-documented (Meara et al., 1999; Patrick et al., 2001). Furthermore, the relationship between PD and similar conditions also accompanied by hand tremor, such as Essential Tremor (ET), is still unclear (Jimenez-Jimenez et al., 2012).

A solution to minimize subjectivity is to introduce sensor-based measurements (Chaudhry et al., 2006), which provide a reproducible and objective assessment of hand tremor and bradykinesia. The Leap Motion Controller (LMC) has been proposed for this task (Garcia-Agundez et al., 2019). Capturing hand movements via contactless sensors has the potential to reduce ambiguity, providing neurologists with more objective assessments of hand dexterity that may lead to more accurate therapeutic adjustments. At the same time, variables that provide meaningful information for the estimation of UPDRS scores could be used to establish more objective assessment scales, allowing for a finer resolution in hand dexterity assessment and better adjusted pharmacologic therapies.

The goal of this article is to provide a systematic review of recent advances in hand dexterity assessment using contactless sensors in PD patients, in the domains of hand tremor and bradykinesia. This review aims to provide further insight into the feasibility and reliability of this paradigm, as well as suggesting best practice guidelines for both engineers and clinicians on how to proceed from this point.

## 2. Methods

As a basis for this systematic review, we searched the databases Pubmed, ScienceDirect, IEEE Xplore, and Cochrane for articles matching the search query:

*(Parkinson* OR*Tremor)* AND*(Leap Motion* OR*Contactless* OR*Infared* OR*Lidar)*

on March 31, 2021. This search yielded the following results:

- 28 matches in Pubmed
- 168 in ScienceDirect, including 10 duplicates
- 18 in IEEE Xplore, including 5 duplicates
- 3 in Cochrane, including 3 duplicates

The search was complemented by seven additional articles selected from the references of search matches, yielding 224 abstracts for screening. The abstracts of these matches were filtered according to the following criteria:

1. Research articles
2. Related to PD
3. Related to hand tremor or bradykinesia

This filtering reduced the abstracts to 47 full-text articles assessed for eligibility. These full-text articles were selected for analysis if they met the following *inclusion criteria*:

1. Articles presenting a method to measure hand tremor or bradykinesia using a contactless approach
2. In patients with PD
3. Aiming to link sensor data to clinical functional performance scores (MDS-UPDRS-III or similar)

Conversely, articles were excluded if they met at least one of the following *exclusion criteria*:

1. Articles not related to hand tremor or bradykinesia. Thirteen articles were excluded with this criterion.
2. Articles without participants (technical or otherwise conceptual papers). Seven further exclusions.
3. Articles aiming to test a novel rehabilitation tool or otherwise not linking sensor data to clinical functional performance scores. Nine further exclusions.
4. Articles not using contactless sensors. Three further exclusions.
5. Articles aiming to classify between PD patients and controls exclusively and not to assess symptom severity. Six further exclusions.

**Figure 1:**
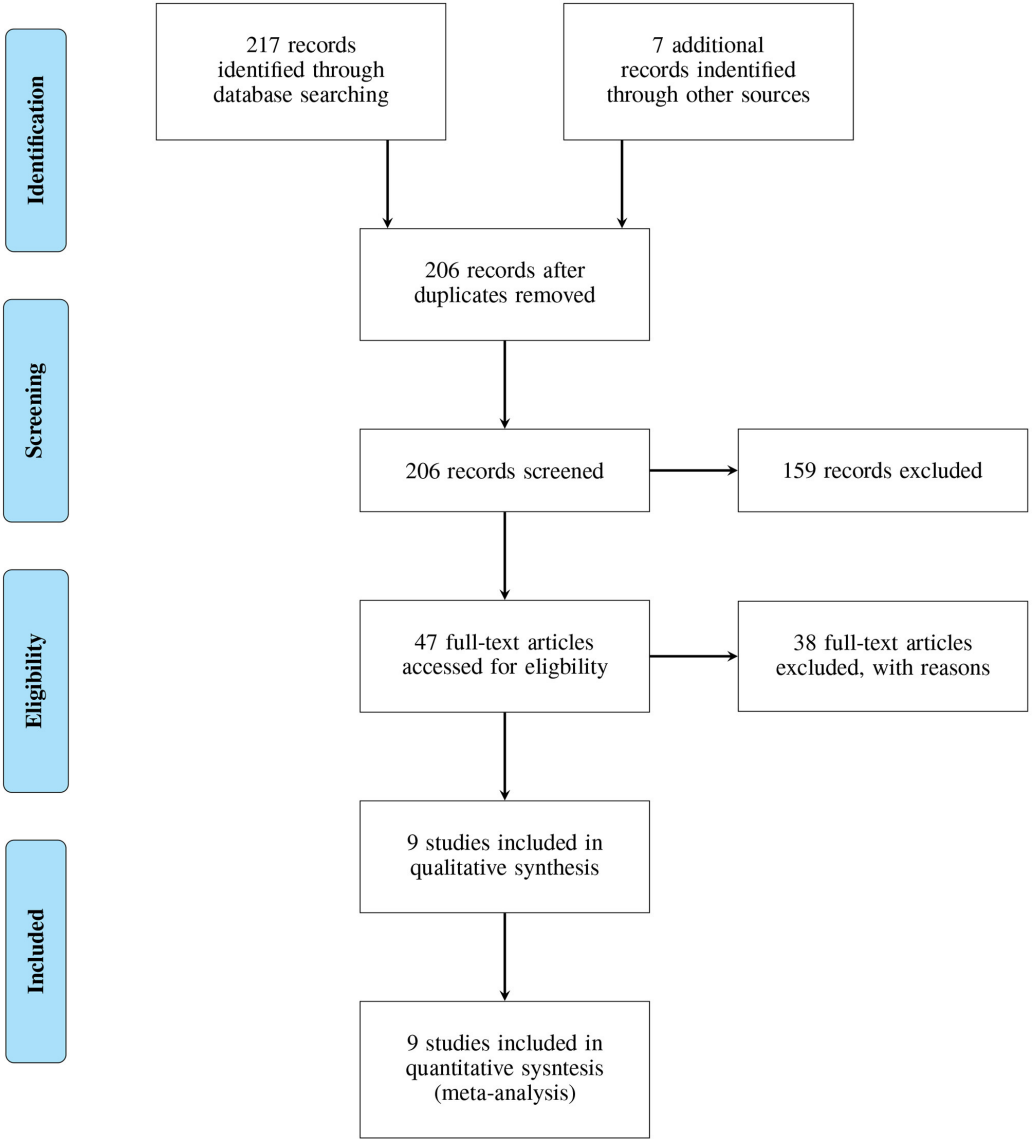
PRISMA 2019 flow diagram.

Finally resulting in *n=9* articles for the qualitative and quantitative analysis (Khan et al., 2014; Butt et al., 2017, 2018; Lugo et al., 2017; Cakmak et al., 2018; Lee et al., 2019; Vivar et al., 2019; Williams et al., 2020a,b). Of the selected articles, six were first identified in the Pubmed search, one in ScienceDirect, and two were selected from the additional articles. This procedure was conducted in accordance with the PRISMA guidelines. A.G. was responsible for the selection and data collection process. The following data were sought from the articles: cohort data, procedure data (assessment method, sensor implementation), classification data, and classification accuracy. No studies are clinical trials and no bias assessment was conducted. The PRISMA flow diagram is included in Figure 1.

## 3. Results

**Figure 2:**
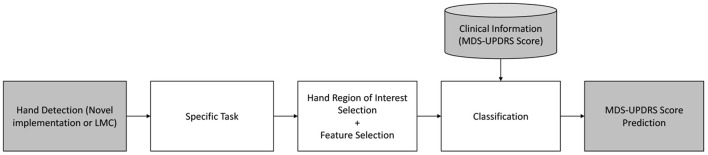
Study design flow diagram.

All identified articles use some form of video source, including hand detection and tracking. Six of the nine articles use the LMC (Butt et al., 2017, 2018; Lugo et al., 2017; Cakmak et al., 2018; Lee et al., 2019; Vivar et al., 2019), while three use other video sources (Khan et al., 2014; Williams et al., 2020a,b). Essentially, all studies follow the same structure: given a dataset of PD patients performing a certain MDS-UPDRS III task (e.g., finger tapping) rated by one or more neurologists and captured with a sensor, the resulting task score (or a linear regression model) is inferred using points of interest of the hand, defined by a series of features, with a classification method, as depicted in Figure 2.

The identified studies implement one or more of the following UPDRS specific tasks:

- Task 3.4, Finger Tapping: The patient taps the index finger on the thumb 10 times as quickly and as big as possible. Out of the nine identified studies, seven analyse this task (Khan et al., 2014; Butt et al., 2017, 2018; Cakmak et al., 2018; Lee et al., 2019; Williams et al., 2020a,b).
- Task 3.5, Hand Movements: The patient makes a tight fist, then opens the hand 10 times as fully and as quickly as possible. Out of the nine studies, three analyse this task (Butt et al., 2017, 2018; Lee et al., 2019).
- Task 3.6, Pronation-Supination: The patient extends the arm with the palm down, then runs the palm up and down alternately 10 times as fast and as fully as possible. The three same studies as above analyse this task (Butt et al., 2017, 2018; Lee et al., 2019).
- Task 3.15, Postural Tremor: The patient stretches the arm with the palms down. Tremor in this posture is observed for 10 s. Out of the nine studies, four analyse this task (Butt et al., 2017, 2018; Lugo et al., 2017; Vivar et al., 2019).
- Task 3.17, Kinetic Tremor: The patient performs at least three finger-to-nose maneuvers. Tremor in this movement is observed. Out of the nine studies, two analyse this task (Lugo et al., 2017; Vivar et al., 2019).

Table 1 summarizes the implemented tasks and signal of interest of each study. With the exception of task 3.5, the choice of hand region of interest for each task is consistent.

**Table 1:** Study tasks and signal of interest.

**References**	**Finger tapping**	**Hand movements**	**Pronation-supination**	**Postural tremor**	**Kinetic tremor**
Khan et al. (2014)	Index finger position				
Vivar et al. (2019)				Palm center × coordinate	Palm center × coordinate
Butt et al. (2017)	Thumb-index fingertip distance	Sum of all palm-fingertip distances	Palm roll angle	Fingertip velocity average	
Butt et al. (2018)	Thumb-index fingertip distance	Sum of all palm-fingertip distances	Palm roll angle	Fingertip velocity average	
Lee et al. (2019)	Thumb-index fingertip distance	Palm-phalanxes median cosine angle	Palm roll angle		
Cakmak et al. (2018)	Thumb-index fingertip distance				
Williams et al. (2020a)	Thumb-index fingertip distance				
Williams et al. (2020b)	Thumb-index fingertip distance				
Lugo et al. (2017)				Palm center coordinates	Palm center coordinates

Table 2 presents the study devices, cohorts, total number, and type of samples, as well as the main study goals. The studies either aim to predict the UPDRS rating of a given sample (Khan et al., 2014; Lugo et al., 2017; Vivar et al., 2019; Williams et al., 2020a) or build a linear regression model relating variables extracted from the signals described in Table 1 and UPDRS scores (Butt et al., 2017, 2018; Cakmak et al., 2018; Lee et al., 2019; Williams et al., 2020b). In this study, we refer to sample as an instance of either hand of a PD patient performing a UPDRS task. Cumulatively, the nine identified studies have a cohort of *n* = 187 patients and 1, 385 samples.

**Table 2:** Study cohorts and goals.

**References**	**Resolution**	**N**	**Sex M/F**	**Age range or Mean (SD)**	**Number of samples**	**Study goals**
Khan et al. (2014)	Camera 352 × 288@25 Hz	13	8/5	50–75	387	Classify UPDRS scores in 0, 1, or 2
Vivar et al. (2019)	LMC@40 Hz	20	11/9	69 (14)	39	Classify UPDRS scores in 0, 1, or 2
Butt et al. (2017)	LMC@35 Hz	16	11/9	68 (7)	96	UPDRS Linear regression
Butt et al. (2018)	LMC@35 Hz	16	11/9	69 (9)	96	UPDRS Linear regression
Lee et al. (2019)	LMC@120 Hz	8	6/2	44-60	144	UPDRS Linear regression
Cakmak et al. (2018)	LMC@100 Hz	24	17/7	57 (9)	378	UPDRS Linear regression
Williams et al. (2020a)	Camera 1,920 × 1,080@60 Hz	20		67 (10)	40	Classify UPDRS scores in < =1 or >1
Williams et al. (2020b)	Camera 1,920 × 1,080@60 Hz	37	24/13	68 (10)	73	UPDRS Linear regression
Lugo et al. (2017)	LMC@40 Hz	33	21/12	65 (12)	132	Classify UPDRS scores in 0, 1, 2, or 3

In the following, we divide the qualitative analysis into two subsections. Section 3.1 compares the results of studies that aim to evaluate the scores of UPDRS tasks related to tremor, 3.15 (Postural Tremor) and/or 3.16 (Kinetic Tremor), while section 3.2 compares the results of studies that aim to evaluate bradykinesia with Tasks 3.4 (Finger Tapping), 3.5 (Hand Movements), and 3.6 (Pronation-Supination).

### 3.1. Tremor

Four studies aimed to assess hand tremor in PD using contactless sensors (Butt et al., 2017, 2018; Lugo et al., 2017; Vivar et al., 2019). All studies used the LMC and either the center of the palm (Lugo et al., 2017; Vivar et al., 2019) or changes in fingertip velocity (Butt et al., 2017, 2018). Butt et al. suggest the use of a 14 Hz lowpass filter, which should not affect the detection of Parkinsonian tremors. The studies also differed greatly in the choice of variables, as well as in the resulting accuracy, if classification was attempted.

Vivar et al. (2019) proposed the use of histogram-based variables, computing an addition and subtraction of data points within a sliding window of 449 samples that advances through the data. Standard features are then computed from these histograms, with contrast and homogeneity providing the best performance. This yielded the best performance in this task group, with an accuracy over 97% classifying scores of 0, 1, and 2.

Lugo et al. (2017) performed a similar study, using a significantly shorter windowing of 15 frames, as well as a different choice of variables. The resulting performance was worse at 74%, albeit the sample size was larger and a patient with a score of 3 on both hands was included.

Finally, Butt et al. (2017, 2018) did not aim to estimate UPDRS scores but rather find variables correlated with said scores. The first study found no correlations between the chosen variables (signal strength and power in the 8–12 Hz band). The second study used the same variables and identified a correlation of *R* = 0.59 with signal strength.

Table 3 summarizes the differences in these studies. Overall, data indicate that detecting resting tremor is feasible, but kinetic tremor is more difficult to identify.

**Table 3:** Classification results for tremor.

**References**	**Signal of Interest**	**Signal preprocessing**	**Variables**	**Results**
Vivar et al. (2019)	Palm center × coordinate	None	Sum and difference of histograms	Bagged Tree classifier, 97% accuracy
Butt et al. (2017)	Fingertip velocity average	14 Hz Lowpass	8–12 Hz Power spectral density, signal strength	No significant correlations found
Butt et al. (2018)	Fingertip velocity average	14 Hz Lowpass	8–12 Hz Power spectral density, signal strength	*R* = 0.59 for signal strength
Lugo et al. (2017)	Palm center coordinates	15-frame windowing	Square Euclidean and Chi Square distance, Earth Mover's distance, Manhattan distance, Shannon entropy, Log energy enthropy	Unspecified classifier, 73.81% accuracy

### 3.2. Bradykinesia

Seven studies aimed to assess at least one UPDRS task related to bradykinesia using contactless sensors (Khan et al., 2014; Butt et al., 2017, 2018; Cakmak et al., 2018; Lee et al., 2019; Williams et al., 2020a,b). These studies used a mixture of LMC and video, and differed greatly in choice of signals and variables. As all of these studies implemented Task 3.4 (Finger Tapping) but only three included additional tasks (Butt et al., 2017, 2018; Lee et al., 2019). Table 4 summarizes the results for all tasks. The following subsections offer a detailed analysis of each task.

**Table 4:** Classification results for bradykinesia.

**Finger tapping**	**Signal preprocessing**	**Variables**	**Results**
Khan et al. (2014)	Moving average filter, Standard deviation outlier removal	Average of the Cross-Correlation between normalized maxima and minima, total taps, tapping speed, tapping speed variation, differences between first and second half of the task, opening velocity, closing velocity, zero crossing rate, signal energy, facial movements	SVM classifier, 82% accuracy
Butt et al. (2017)	14 Hz Lowpass	Number of repetitions, speeds, variability of frequency and amplitude, power spectral density	Significant correlations for opening speed (*R* = 0.515) and closing speed (*R* = 0.602)
Butt et al. (2018)	14 Hz Lowpass	Number of repetitions, speeds, variability of frequency and amplitude, power spectral density	Significant Correlations for number of repetitions (*R* = 0.728), closing speed (*R* = 0.804) and opening speed (*R* = 0.836)
Lee et al. (2019)	120 Hz Linear interpolation	Amplitudes, frequencies, velocities and slopes	Significant correlations for velocity and frequency (*R* = 0.45), *R* = 0.86 combining all tasks
Cakmak et al. (2018)	None	Mean and standard deviation of speed, acceleration, frequency	root mean square error of 4.37 points (7.8%) for UPDRS-III and 2.12 points (10.7%) for bradykinesia
Williams et al. (2020a)	PCA	Power spectral density, frequency, peaks, ratio of maxima to minima, standard deviation of peaks	SVM classifier, 84% accuracy
Williams et al. (2020b)	Savitzky-Golay	Amplitude, speed, amplitude variability, power spectral density	Significant correlations for speed (*R* = 0.56), amplitude variability (*R* = 0.61) and rhythm regularity (*R* = 0.50), *R* = 0.69 using all variables
**Hand movements**	**Signal preprocessing**	**Variables**	**Hand movements results**
Butt et al. (2017)	14 Hz Lowpass	Number of repetitions, speeds, variability of frequency and amplitude, power spectral density	Significant correlations for variability of frequency (*R* = 0.685) and number of repetitions (*R* = 0.630)
Butt et al. (2018)	14 Hz Lowpass	Number of repetitions, speeds, variability of frequency and amplitude, power spectral density	Significant correlations for opening speed (*R* = 0.647), Variability of amplitude (*R* = 0.647), closing speed (= 0.639) and number of repetitions (*R* = 0.539)
Lee et al. (2019)	120 Hz Linear interpolation	Amplitudes, frequencies, velocities and slopes	Significant correlation for velocity (*R* = 0.69), *R* = 0.86 combining all tasks
**Pronation-supination**	**Signal preprocessing**	**Variables**	**Pronation-supination results**
Butt et al. (2017)	14 Hz Lowpass	Number of repetitions, speeds, variability of frequency and amplitude, power spectral density	Significant correlation for variability of amplitude (*R* = 0.858)
Butt et al. (2018)	14 Hz Lowpass	Number of repetitions, speeds, variability of frequency and amplitude, power spectral density	Significant correlation for variability of frequency (*R* = 0.488)
Lee et al. (2019)	120 Hz Linear interpolation	Amplitudes, frequencies, velocities and slopes	Significant correlation for amplitude (*R* = 0.56), *R* = 0.86 combining all tasks

#### 3.2.1. Task 3.4 (Finger Tapping)

With the exception of Khan et al. (2014), all studies used the Euclidean distance between the tip of the index finger and the thumb as signal of interest. All studies are also reasonably consistent in the choice of variables: number of repetitions, amplitudes, variability of amplitude (particularly a decrease in amplitude with subsequent repetitions), speeds (generally considered as opening and closing speeds separately), accelerations, and frequency domain analysis. We can divide these seven studies into two groups: two that classify UPDRS scores (Khan et al., 2014; Williams et al., 2020a) and five that use linear regression instead (Butt et al., 2017, 2018; Cakmak et al., 2018; Lee et al., 2019; Williams et al., 2020b).

The two studies aiming at classification (Khan et al., 2014; Williams et al., 2020a) used video instead of a LMC. Interestingly, the resolution and frequency employed by Khan et al. (2014) is significantly lower, with a smaller number of participants but a significantly larger number of samples and a more complex classification task, as they aim to classify ternary scores of 0, 1, and 2 instead of classifying scores binarily as ≤ 1 vs. >1 (Williams et al., 2020a). Both obtained the best results when using support vector machines, with overall accuracies of 82% for Khan (Khan et al., 2014) and 84% for Williams (Williams et al., 2020a).

The remaining five studies used linear regression (Butt et al., 2017, 2018; Cakmak et al., 2018; Lee et al., 2019; Williams et al., 2020b). Some, but not all studies report the correlation of each of the variables individually. Overall, correlated variables fall within the [0.5, 0.6] range, with Butt et al. (2018) reporting significantly higher correlations for opening (*R* = 0.836) and closing (*R* = 0.804) speeds. Table 5 provides a direct comparison of the correlations of these studies. Overall, data indicate that assessing UPDRS scores with video is feasible, and opening and closing speeds show good correlations with UPDRS scores.

**Table 5:** Correlation results for bradykinesia.

**Finger tapping**	**Opening speeds**	**Closing speeds**	**Number of repetitions**	**Frequency**	**Amplitude**
Butt et al. (2017)	−0.515	−0.602			
Butt et al. (2018)	−0.836	−0.804	−0.728	−0.006	−0.188
Lee et al. (2019)	0.45^a^	0.45^a^		0.45^a^	
Williams et al. (2020b)	−0.56^b^	−0.56^b^		−0.5	0.61
**Hand movements**	**Opening speeds**	**Closing speeds**	**Number of repetitions**	**Frequency**	**Amplitude**
Butt et al. (2017)			−0.63	−0.685	
Butt et al. (2018)	−0.647	−0.639	−0.539	0.313	−0.647
Lee et al. (2019)	0.69^b^	0.69^b^			
**Pronation-supination**	**Opening speeds**	**Closing speeds**	**Number of repetitions**	**Frequency**	**Amplitude**
Butt et al. (2017)					−0.858
Butt et al. (2018)	−0.009	−0.025	−0.257	−0.488	0.307
Lee et al. (2019)					0.56

*Amplitude refers to amplitude or variations thereof. Frequency refers to frequency or variations thereof*.

a *Combination of all elements*.

b *No distinction between opening and closing speeds*.

#### 3.2.2. Task 3.5 (Hand Movements)

Concerning Task 3.5 (Hand Movements), no classification has been implemented yet. Lee et al. (2019) explored the correlation of a 120 Hz linearly interpolated signal analyzed through amplitudes, frequencies, velocities, and slopes. The number of participants was small (eight), but a large number of samples was collected by measuring with and without deep brain stimulation. They employed the angle between the fingers and the palm as signal of interest. As they did not explore the regression coefficients on each task individually but rather build a global linear regression model, only the velocity of Task 3.5 is reported as showing a relevant correlation of *R* = 0.69.

Butt et al. (2017, 2018) also implemented this task in their two studies, using the Euclidean distance between palm and fingertips. Again employing a 14 Hz lowpass filter, they explored a very similar set of variables, using number of repetitions, speeds, the variability of frequency and amplitude, and power spectral density. They do report the individual correlation of each of the explored variables, showing significant correlations in most variables. Interestingly, the correlations vary substantially between both studies.

Table 5 offers a comparison between the correlations of these three studies. Overall, data indicate good correlations for opening and closing speeds. No study has attempted to classify UPDRS scores so far.

#### 3.2.3. Task 3.6 (Pronation-Supination)

The same three studies as in the previous subsection implemented Task 3.6, using the same variables as in the previous task but focusing on a different point of the hand, the roll angle of the palm. All three studies report worse results with Task 3.6, as summarized in Table 5. Overall, data only shows good correlations for amplitude and variability of amplitude. No study has attempted to classify UPDRS scores so far.

## 4. Discussion

In this systematic review, we analyzed recent advances in sensor-based, UPDRS-inspired tremor and bradykinesia assessment in PD patients.

Concerning tremor, it seems that the coordinates of the palm center are a good predictor of UPDRS scores. Larger windows as well as statistical variables seem to be a better choice. Although the studies did not include patients with higher scores (three and four) classifying these should be easier as tremor is expected to be more severe. Although the limited number of studies does not yield definite conclusions, it would seem that classifying tremor UPDRS scores is nearly as accurate as classifying PD patients and healthy controls.

**Figure 3:**
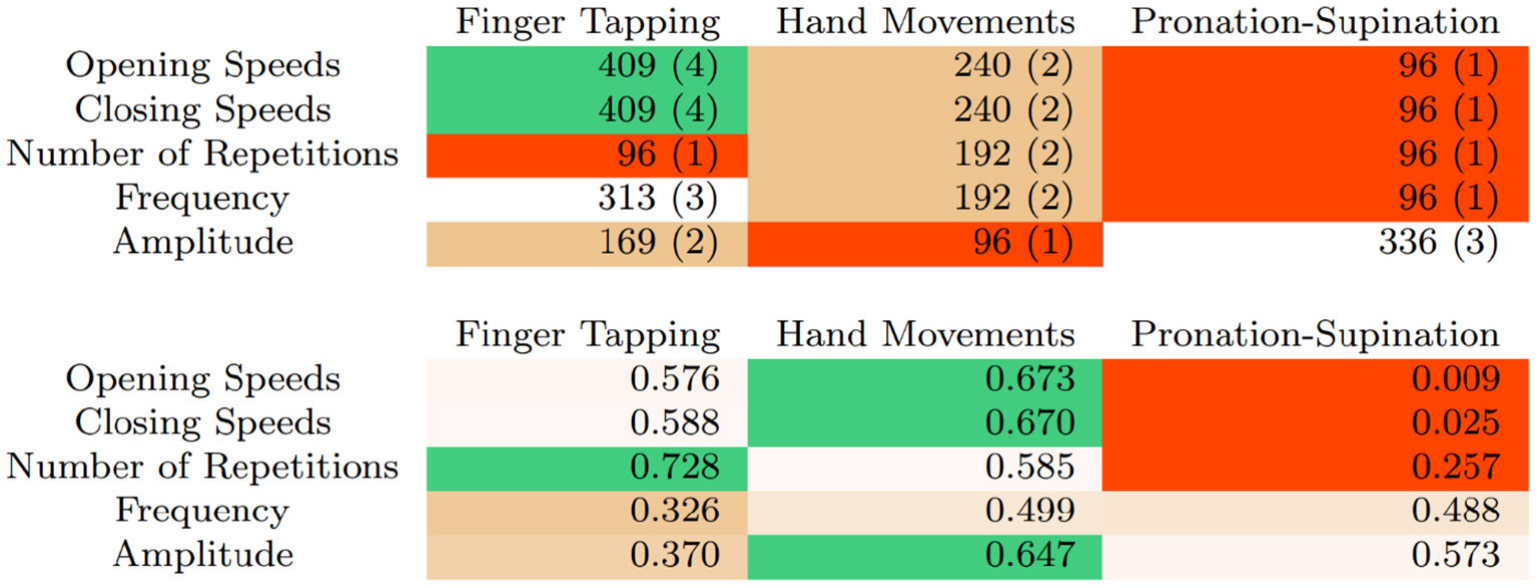
Number of samples and (number of articles) using different variables and UPDRS bradykinesia tasks **(top)** and sample-weighted correlations **(bottom)**.

Figure 3 summarizes the number of samples, studies and sample-weighted correlations of all UPDRS bradykinesia tasks. The number of repetitions, opening and closing speeds, combined with changes in amplitude as the task progresses, seem to best characterize the rating in Task 3.4 (Finger Tapping). Implemented classification schemes in this scenario can already achieve excellent results, with accuracies over 80% when discriminating scores of 0, 1, and 2. As is the case with tremor, including higher scores would probably not decrease accuracy as these represent patients that are either almost (3) or fully (4) incapable of performing the task.

For Tasks 3.5 (Hand Movements) and 3.6 (Pronation-Supination) no full classification has been implemented yet. Early results seem to suggest that this task is more difficult to rate, as correlations between variables and neurologist ratings are somewhat lower, in the 0.5–0.6 range. A significant exception is variability of amplitude, which seemed to perform better in Butt et al. (2017).

In this study, we limited ourselves to contactless sensors because we believe the advantages of this approach are significant. However, contactless sensors cannot provide a comprehensive method to measure and quantify all motor symptoms of PD, since they cannot assess the stiffness and rigidity of the arms and legs. They are also more limited than electromyography, which provides richer information on muscular activity. On the positive side, they do not require any adjustment to the patient or any interaction other than the performance of the manual tasks, providing an ideal setup to monitor some of the motor symptoms of PD remotely as an addition, rather than a substitution, of more comprehensive PD assessment methods. Other contactless approaches, such as e.g., Lidar, remain to be explored. Finally, the LMC also presents the additional limitations of infrared sensors, such as measurement noise. Numerous authors indicate that the LMC is fallible depending on environmental light and dirt on the lens being present.

In spite of the limitations of this study, and considering the number of relevant studies is still small, available early evidence points to the LMC offering a feasible, objective alternative to visual observation to capture and rate some features of hand motility in PD, as well as in other related diseases. Evidence shows that sensor-based methods have clinical potential and might, after refinement, complement, or even replace subjective assessment procedures, not only in patient care but as an additional outcome measure in the clinical trials of disease-modifying treatments. A significant advantage of a sensor-based approach is that a linear regression model could provide a much higher resolution than current UPDRS assessment. Apart from this advantage, a sensor-based assessment also shows potential to link objective tremor and bradykinesia assessment to dopaminergic replacement therapy (DRT) dosage directly. In this sense, a more accurately adjusted dose might help maximize the period in which DRT is effective as dosage needs to be subsequently increased and OFF periods become longer.

Nevertheless, a substantial number of additional studies in several domains are required. Future research should focus on including more than one clinician rating, as well as procedure standardization. Once pilot trials achieve UPDRS classification predictions that fall within the inter-rater range, designing expert systems that offer a much finer resolution of tremor and bradykinesia should become feasible.

## Data Availability Statement

The original contributions presented in the study are included in the article/supplementary material, further inquiries can be directed to the corresponding author/s.

## Author Contributions

Both authors listed have made a substantial, direct, and intellectual contribution to the work, and approved it for publication.

## Conflict of Interest

The authors declare that the research was conducted in the absence of any commercial or financial relationships that could be construed as a potential conflict of interest.

## Publisher's Note

All claims expressed in this article are solely those of the authors and do not necessarily represent those of their affiliated organizations, or those of the publisher, the editors and the reviewers. Any product that may be evaluated in this article, or claim that may be made by its manufacturer, is not guaranteed or endorsed by the publisher.
